# Perspectives on the COVID-19 pandemic impact on cardio-oncology: results from the COVID-19 International Collaborative Network survey

**DOI:** 10.1186/s40959-020-00085-5

**Published:** 2020-11-27

**Authors:** Diego Sadler, Jeanne M. DeCara, Joerg Herrmann, Anita Arnold, Arjun K. Ghosh, Husam Abdel-Qadir, Eric H. Yang, Sebastian Szmit, Nausheen Akhter, Monika Leja, Carolina Maria Pinto Domingues Carvalho Silva, Jayant Raikhelkar, Sherry-Ann Brown, Susan Dent, Rupal O’Quinn, Franck Thuny, Rohit Moudgil, Luis E. Raez, Tochukwu Okwuosa, Andres Daniele, Brenton Bauer, Lavanya Kondapalli, Roohi Ismail-Khan, Jorge Lax, Anne Blaes, Zeina Nahleh, Leah Elson, Lauren A. Baldassarre, Vlad Zaha, Vijay Rao, Daniel Sierra Lara, Kerry Skurka, on behalf of the Cardio-Oncology International Collaborative Network

**Affiliations:** 1 Heart and Vascular Center, Cleveland Clinic Florida, 2950 Cleveland Clinic Blvd, Weston, FL 33331 USA; 2 University of Chicago, Chicago, USA; 3 Mayo Clinic, Rochester, MN USA; 4 Lee Health, Ft. Myers, USA; 5 Barts Heart Centre, St Bartholomew’s Hospital, and University College London’s Hospital, London, UK; 6 Women’s College Hospital and Peter Munk Cardiac Centre, University of Toronto, Toronto, Canada; 7 UCLA Cardio-Oncology Program, University of California, Los Angeles, USA; 8 Centre of Postgraduate Medical education, Warsaw, Poland; 9 Northwestern Feinerg School of Medicine, Chicago, USA; 10 University of Michigan, Ann Arbor, MI USA; 11 University of Sao Paulo, Sao Paulo, Brazil; 12 Columbia University Irving Medical Center, New York, NY USA; 13 Medical College of Wisconsin, Milwaukee, WI USA; 14 Duke University, Durham, NC USA; 15 University of Pennsylvania, Philadelphia, PA USA; 16 Aix-Marseille University, Marseille, France; 17 Cleveland Clinic, Cleveland, OH USA; 18 Memorial Health Care, Florida International University, Miami, FL USA; 19 Rush University Medical Center, Chicago, IL USA; 20 Roffo Institute, Buenos Aires, Argentina; 21 Torrance Memorial Medical Center, Torrance, CA USA; 22 University of Colorado, Aurora, CO USA; 23 H. Lee Moffitt Cancer Center, Tampa, FL USA; 24 Hospital Cosme Argerich, Buenos Aires, Argentina; 25 University of Minnesota, Minneapolis, MN USA; 26 Yale School of Medicine, New Haven, CT USA; 27 UT Southwestern, Dallas, TX USA; 28 Franciscan Health, Indianapolis, IN USA; 29 Instituto Nacional de Cardiologia, Ciudad de Mexico, Mexico

**Keywords:** COVID-19, Health policy, Global Health, Cardio oncology

## Abstract

**Background:**

Re-allocation of resources during the COVID-19 pandemic has resulted in delays in care delivery to patients with cardiovascular disease and cancer. The ability of health care providers to provide optimal care in this setting has not been formally evaluated.

**Objectives:**

To assess the impact of COVID-19 resource re-allocation on scheduling, testing, elective procedures, telemedicine access, use of new COVID-19 therapies, and providers’ opinions on healthcare policies among oncology and cardiology practitioners.

**Methods:**

An electronic survey was conducted by a cardio-oncology collaborative network through regional and state chapters of the American College of Cardiology, American Society of Clinical Oncology, and the International Cardio-Oncology Society. Descriptive statistics were reported by frequency and proportion for analyses, and stratified categorically by geographic region and specialty.

**Results:**

One thousand four hundred fifteen providers (43 countries) participated: 986 cardiologists, 306 oncologists, and 118 trainees/internal medicine. 63% (195/306) of oncologists vs 92% (896/976) of cardiologists reported cancellations of treatments/elective procedures (*p* = 0.01). 46% (442/970) of cardiologists and 25% (76/303) of oncologists modified the scope of their practice (*p* = < 0.001). Academic physicians (74.5%) felt better supplied with personal protective equipment (PPE) vs non-academic (74.5% vs 67.2%; *p* = 0.018). Telemedicine was less common in Europe 81% (74/91), and Latin America 64% (101/158), than the United States, 88% (950/1097) (*p* = < 0.001). 95% of all groups supported more active leadership from medical professional societies.

**Conclusions:**

These results support initiatives to promote expanded coverage for telemedicine, increased access to PPE, better testing availability and involvement of medical professional societies to help with preparedness for future health care crisis.

**Supplementary Information:**

**Supplementary information** accompanies this paper at 10.1186/s40959-020-00085-5.

## Introduction

The coronavirus disease 2019 (COVID-19) pandemic has resulted in substantial morbidity and mortality across the world, with over 30 million cases, and 1 millon deaths worldwide, as of late September 2020 [1]. Almost every country has now been significantly impacted. With this, there has been a massive shift of medical resources focusing on the testing and treatment of COVID-19 patients, resulting in delays of other, non-COVID-19-related medical care, including that required for cardiovascular diseases (CVD) and cancer, the two leading causes of death in the western world [2].

Importantly, patients with pre-existing CVD and cancer are particularly vulnerable to the severe acute respiratory syndrome corona virus 2 (SARS CoV-2), the agent responsible for the COVID-19 pandemic, with increased morbidity and mortality [3–7]. In addition to the direct infliction by COVID-19, these patient population are severely affected in an indirect manner by the change in health care resource allocations. These include rescheduling or postponing of cardiac testing, procedures, advanced imaging, and cancer treatments, and the effects of these are emerging [8–11]. Of those directly caring for patients with COVID-19, efforts were at times thwarted by critical supply shortages leading to inadequate testing and PPE, further complicated by, at times, tepid institutional support [12–17]. The impact of the COVID 19 pandemic on subgroups of health care providers has recently been reported [18, 19], but cardiologists’ and oncologists’ opinions and needs throughout this global health crisis have not been formally evaluated. Such data are important to foster a better understanding of the impact and future preparedness of healthcare crises. The current survery was conducted among cardiologists and oncologists based on a Cardio-Oncology collaborative network with members of regional and state Chapters of the American College of Cardiology (ACC), American Society of Clinical Oncology (ASCO) and the International Cardio Oncology Society (ICOS). The summary results and recommendations from this survery are reported herein.

## Methods

We conducted a survey between March 24th and April 17th 2020 to measure the impact of the pandemic on provider practices and the availability of resources worldwide. A link to the electronic survey (Apendix) was sent via e mail by local professional societies on behalf of the ACC/ASCO/ICOS State Chapter collaborative network, supported by members of 19 states within the United States (US) and 10 countries. E-mail reminders were sent to potential responders on the second and third week by the participating sites. The Survey had 20 questions, 7 collected demographics and 13 questions collected information about resources, treatments, institutional support and opinions on the COVID 19 pandemic response. The survey targeted primarily adult cardiologists and oncologists but also included a small number of internal medicine physicians. We compared the responses of cardiologists versus oncologists, academic versus non academic practices, and the respondents by geographic area. Participants received no incentives or compensation. No personal, health, or protected information was obtained with this survey.

All responses were included for analysis, except for geographic comparisons where only North America, Europe and Latin America were included given the small numbers from other locations. The Florida Chapter of the ACC collected the electronic survey data. Data was directly and securely exported to Microsoft Excel (2010). Descriptive statistics were reported via frequency and proportion for analyses, and stratified categorically by geographic region and specialty. Chi square analysis was performed to establish statistical differences between categorical variables, and two-tailed *p*-values were reported. Bonferroni correction was used for comparisons between three groups. There were no continuous variables in this survey. Statistical significance was interpreted based on an α-value < 0.05. All data analyses were performed using SPSS, version 26 (IBM, Aramonk, NY).

## Results

### Demographics

**Fig. 1:**
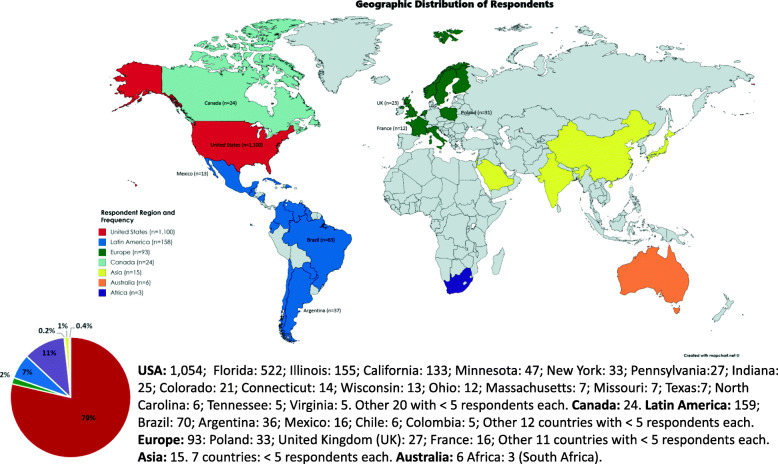
Distribution of Respondents: World map displaying color-coded regions wherein survey respondents to the international survey are currently practicing. Countries with > 5 respondents are listed, with their respective respondent numbers

There were 1415 respondents to the survey from 43 countries: By global region, 1124 were from North America (US and Canada), 158 from Latin America, 93 from Europe, 15 from Asia, 6 from Australia and 3 from Africa. The geographic distribution of respondents is depicted in the Fig. 1.

**Fig. 2:**
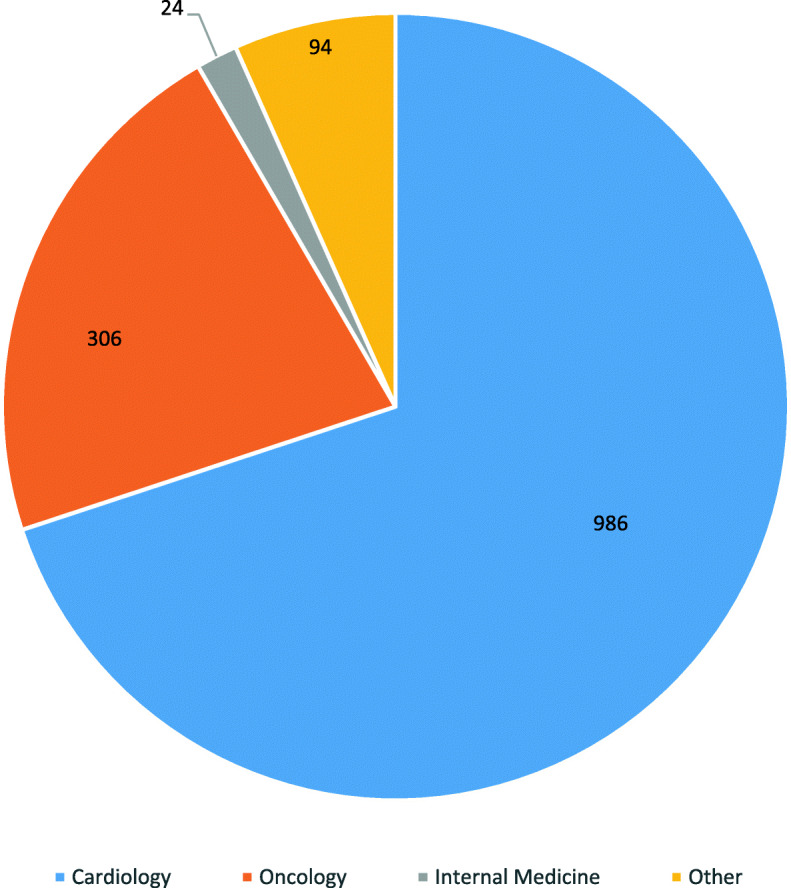
Reported Medical Specialties: Distribution of respondents, per self-identified medical specialty: includes cardiology, oncology, internal medicine, and other. Most respondents practiced cardiology, followed by oncology

Among the respondents, there were 986 cardiologists, 306 oncologists, and 118 classified as others including trainees and internal medicine providers (Fig. 2). Gender representation varied by specialty. Sixty-five percent (642/ 986) of the cardiology respondents were males but 67% (207/306) of the oncology respondents were females (*p* = < 0.001). A hospital-based practice was reported by 40% of both cardiologists and oncologists. Thirty percent (298/980) of cardiologists and 41% (127/306) of oncologists were working in academic settings.

### Impact of pandemic in practice patterns including scheduling, testing, procedures

**Fig. 3:**
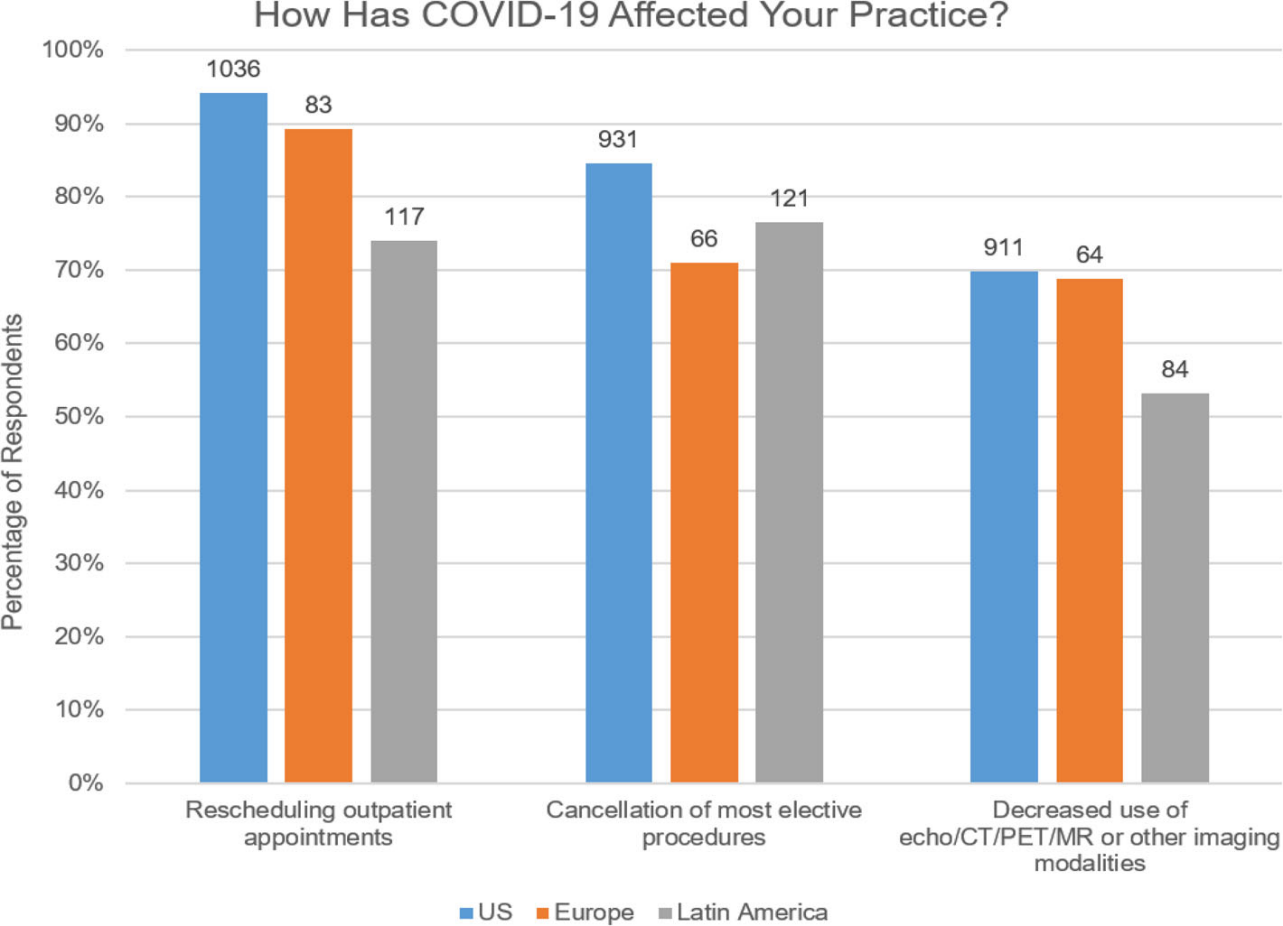
How Has COVID Affected Your Practice? Impact of COVID-19 in practice patterns, stratified by geographic region

Ninety-two percent (1292/1402) of survey participants reported rescheduling patients as a result of the pandemic, a response that was remarkably similar across all surveyed groups and locations. Ninety-two percent (896/976) of cardiologists versus 63% (195/306) of oncologists reported cancellations of elective procedures/day-unit cancer outpatient treatment due to the pandemic (*p* = 0.01). Similarly, decreased use of diagnostic cardiovascular (CV) imaging was reported by 81% (791/976) of cardiologists but only 39% (120/305) of oncologists (*p* = < 0.001). These diagnostic modalities were cancelled in 71% (911/1281) of physician practices in the US, 53% (84/158) in Latin America and 69% (64/93) in Europe at the time of the survey (Fig. 3).

Oncology treatment modification to balance increased risk of exposure was reported by 72% (221/305) of oncologists.

### Deployment of physicians

Forty-six percent (442/970) of cardiologists but only 25% (76/303) of oncologists were asked to modify the scope of their specialized practice and redeploy from specialty care to help with acute patient care during the initial phase of the COVID-19 health care crisis (*p* = < 0.001) (Table 1). The change of scope of practice was higher in non US locations: 96/156 (62%) of Latin America and 61/93 (66%) of European providers as opposed to 368/1081 (34%) of US providers reported a temporary change in their scope of practice (*p* = < 0.001) (Table 3).

**Table 1:** Responses: cardiologists vs oncologists; *p*-values determined via chi-squared analysis

Survey Question	Cardiologists		Oncologists		***P***-value
	Yes (n (%))	No (n (%))	Yes (n (%))	No (n (%))	Statistical significance < 0.05
“Does your institution provide proper protection for all health care workers to the level they are exposed?”	684 (70.6%)	285 (29.4%)	210 (69.3%)	93 (30.7%)	*P* = 0.598
“Have timely surgery/chemo/immunotherapy treatments for your patients been delayed since the COVID-19 pandemic started?”	498 (56.4%)	385 (43.6%)	152 (50%)	152 (50%)	*P* < 0.001
“Do you use telemedicine to reduce in-person encounters and health care providers’ exposure?”	818 (85.0%)	151 (15.0%)	269 (89.4%)	32 (10.6%)	*P* = 0.085
“Have you been asked to reduce your specialized practice to help/contribute to other areas where urgent help is needed?”	442 (45.5%)	529 (54.5%)	76 (25.2%)	226 (74.8%)	*P* < 0.001
“Have you had in service instruction/guidelines from leadership at your institution about policies to protect your patients, your team, your colleagues and yourself?”	839 (86.1%)	136 (13.9%)	272 (89.2%)	33 (10.8%)	*P* = 0.168
“Do you feel you have adequate support from your institution’s leadership to carry on with your duties?”	690 (71.5%)	275 (28.5%)	232 (76.1%)	73 (23.9%)	*P* = 0.030
“Do you discuss with all your patients the importance of strict adherence to COVID-19 community behavior as part of your office/clinic encounters?”	885 (91.5%)	82 (8.5%)	285 (95%)	15 (5%)	*P* = 0.141
“Do you have access to COVID-19 testing at your institution or local lab?”	730 (75.1%)	242 (24.9%)	205 (67.4%)	99 (32.6%)	*P* = 0.017
“Has your institution used Tocilizumab or other anti-inflammatory agents for COVID-19 related myocarditis?”	130 (15.1%)	733 (84.9%)	42 (16.7%)	210 (83.3%)	*P* = 0.059
Has your institution used empiric treatment with Remdesivir or other anti-viral treatments for the sickest patients?	289 (32.7%)	595 (67.3%)	69 (26.1%)	195 (73.9%)	*P* = 0.035
“In your opinion, should there be a government mandated nationwide quarantine/lock-down to slow down the propagation/transmission of COVID-19?”	826 (85.7%)	138 (14.3%)	259 (86.6%)	40 (13.4%)	*P* = 0.909
“Should medical professional organizations take a more active role, and have more influence in official health care policy decisions during this major crisis?”	926 (95.2%)	47 (4.8%	289 (95.7%)	13 (4.3%)	*P* = 0.931

### Available physician resources during the pandemic

#### PPE

Concern about PPE was reported by both cardiologists and oncologists. Overall, physicians reported that their institutions provided proper protection to all healthcare workers for 66% (644/974) of the respondents in March, and to 78.5% (314/400) in April. Physicians practicing in academic institutions felt better supplied with PPE. For instance, 74.4% (360/483) of academic vs 67.4% (611/909) of non-academic private practice and hospital based doctors felt their institutions provided proper PPE (*p* = 0.018) (Table 2). Overall use of PPE was 70.3% (759/1079) in the US vs. 62.4% (58/93) in Europe and 66.2% (104/157) in Latin America (*p* = 0.168).

**Table 2:** Academic versus non academic setting; *p*-values determined by chi-squared analysis

Survey Question	Academic		Non-Academic		***P***-value
	Yes (n (%))	No (n (%))	Yes (n (%))	No (n (%))	Statistical significance < 0.05
“Does your institution provide proper protection for all health care workers to the level they are exposed?”	360 (74.4%)	124 (25.6%)	611 (67.4%)	296 (32.7%)	0.018
“Have timely surgery/chemo/immunotherapy treatments for your patients been delayed since the COVID-19 pandemic started?”	216 (47.5%)	239 (52.5%)	479 (56.8%)	364 (43.2%)	0.157
“Do you use telemedicine to reduce in-person encounters and health care providers’ exposure?”	438 (90.3%)	47 (9.7%)	727 (80.2%)	180 (19.8%)	< 0.001
“Have you been asked to reduce your specialized practice to help/contribute to other areas where urgent help is needed?”	196 (40.4%)	289 (59.6%)	356 (39.3%)	551 (60.7%)	0.325
“Have you had in service instruction/guidelines from leadership at your institution about policies to protect your patients, your team, your colleagues and yourself?”	451 (92.6%)	36 (7.4%)	765 (83.9%)	148 (16.2%)	< 0.001
“Do you feel you have adequate support from your institution’s leadership to carry on with your duties?”	391 (80.3%)	96 (19.7%)	609 (67.5%)	293 (32.5%)	< 0.001
“Do you discuss with all your patients the importance of strict adherence to COVID-19 community behavior as part of your office/clinic encounters?”	436 (90.5%)	46 (9.5%)	837 (92.7%)	66 (7.3%)	0.199
“Do you have access to COVID-19 testing at your institution or local lab?”	407 (84.1%)	77 (15.9%)	607 (66.7%)	303 (33.3%)	< 0.001
“Has your institution used Tocilizumab or other anti-inflammatory agents for COVID-19 related myocarditis?”	93 (22.5%)	320 (77.5%)	93 (11.5%)	714 (88.5%)	< 0.001
Has your institution used empiric treatment with Remdesivir or other anti-viral treatments for the sickest patients?	166 (38.6%)	264 (61.4%)	215 (26.1%)	609 (73.9%)	< 0.001
“In your opinion, should there be a government mandated nationwide quarantine/lock-down to slow down the propagation/transmission of COVID-19?”	421 (88.1%)	57 (11.9%)	766 (84.5%)	140 (15.5%)	0.186
“Should medical professional organizations take a more active role, and have more influence in official health care policy decisions during this major crisis?”	467 (96.5%)	17 (3.5%)	861 (94.6%)	49 (5.4%)	0.209

#### Testing

**Fig. 4:**
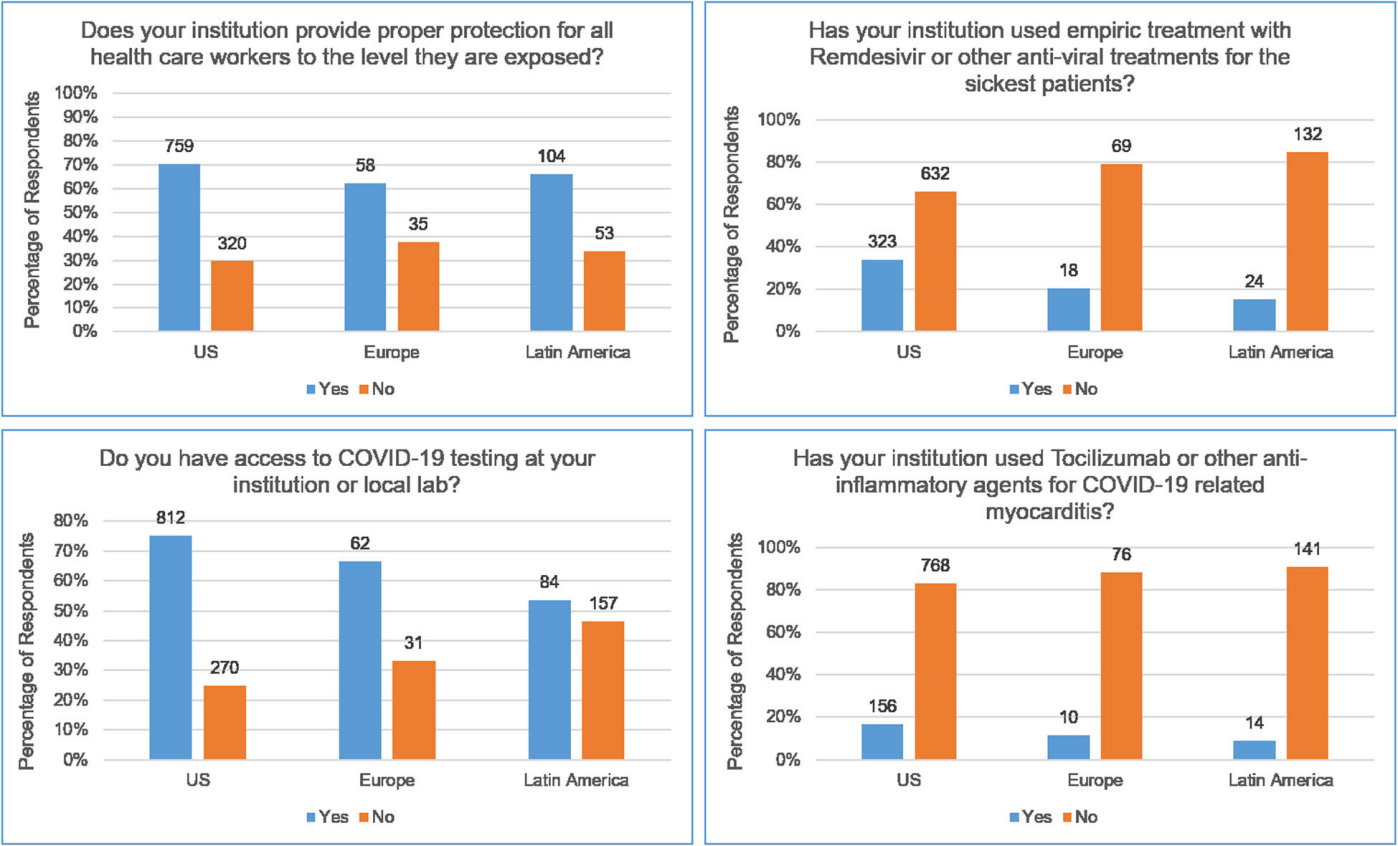
PPE, Testing, and New Treatment Availability. Distribution of practices patterns and treatment types utilized in three geographic regions including, the US, Latin America, and Europe. There use a low use rate for Remdesivir or Tocilizumab, across all three regions; the majority of respondents report availability of PPE and COVID testing, on-site, across all three regions

At the time of the survey, 75% (730/972) of cardiologists and 67% (205/305) of oncologists reported having direct access to COVID-19 testing at their facilities or local labs (*p* = 0.017). (Table 1). The availability of COVID-19 testing also had significant geographic variation: 75% (812/1082) in the US, 67% (62/93) in Europe, and 53.5% (84/157) in Latin America (*p* = < 0.001) (Fig. 4). Direct access to COVID-19 testing was available to 84.1% (407/484) of doctors working in an academic setting but only 66.7% (607/910) of non-academic affiliated doctors, including those in private practice and hospital-employed (*p* = < 0.001) (Table 2).

#### Telemedicine

More than 85% of both cardiologists and oncologists adopted telemedicine during the pandemic, and the use of telemedicine was reported by 82% (798/973) of respondents in March and by 91.5% (366/400) in April. Telemedicine was more commonly used amongst doctors in academic settings 90.3% (438/484) vs non- academic affiliated doctors 80.2%, (727/907) (*p* = < 0.001) (Table 2). The use of telemedicine was less common in Europe and Latin America, 81% (74/91) and (64%) (101/158), respectively, compared to the US where it was 88% (950/1097) (*P* = 0.021 for Europe vs. US; *P* < 0.001 for Latin American vs. US) (Table 3).

**Table 3:** Responses by geographic area; *p*-values determined via chi-squared analysis with multiple group comparison

Survey Question	United States		Europe		Latin America		*P*-value
	Yes (n (%))	No (n (%))	Yes (n (%))	No (n (%))	Yes (n (%))	No (n (%))	Statistical significance < 0.05
“Does your institution provide proper protection for all health care workers to the level they are exposed?”	759 (70.3%)	320 (29.7%)	58 (62.4%)	35 (37.6%)	104 (66.2%)	53 (33.8%)	0.168
“Have timely surgery/chemo/immunotherapy treatments for your patients been delayed since the COVID-19 pandemic started?”	565 (56.8%)	429 (432%)	44 (50%)	44 (50%)	87 (56.1%)	68 (43.9%)	0.009
“Do you use telemedicine to reduce in-person encounters and health care providers’ exposure?”	950 (88%)	129 (12%)	74 (81.3%)	17 (18.7%)	101 (63.9%)	57 (36.1%)	< 0.001
“Have you been asked to reduce your specialized practice to help/contribute to other areas where urgent help is needed?”	368 (34%)	713 (66%)	61 (65.6%)	32 (34.4%)	96 (61.5%)	60 (38.5%)	< 0.001
“Have you had in service instruction/guidelines from leadership at your institution about policies to protect your patients, your team, your colleagues and yourself?”	943 (86.7%)	145 (13.3%)	83 (89.2%)	10 (10.8%)	132 (84.1%)	25 (15.9%)	0.609
“Do you feel you have adequate support from your institution’s leadership to carry on with your duties?”	800 (74.1%)	279 (25.9%)	56 (60.2%)	37 (39.8%)	96 (61.9%)	59 (38.1%)	0.001
“Do you discuss with all your patients the importance of strict adherence to COVID-19 community behavior as part of your office/clinic encounters?”	978 (91.1%)	95 (8.9%)	87 (94.6%)	5 (5.4%)	152 (96.2%)	6 (3.8%)	0.035
“Do you have access to COVID-19 testing at your institution or local lab?”	812 (75%)	270 (25%)	62 (66.7%)	31 (33.3%)	84 (53.5%)	73 (46.5%)	< 0.001
“Has your institution used Tocilizumab or other anti-inflammatory agents for COVID-19 related myocarditis?”	156 (16.9%)	768 (83.1%)	10 (11.6%)	76 (88.4%)	14 (9%)	141 (91%)	< 0.001
Has your institution used empiric treatment with Remdesivir or other anti-viral treatments for the sickest patients?	323 (33.8%)	632 (66.2%)	18 (20.7%)	69 (79.3%)	24 (15.4%)	132 (84.6%)	< 0.001
“In your opinion, should there be a government mandated nationwide quarantine/lock-down to slow down the propagation/transmission of COVID-19?”	910 (84.4%)	168 (15.6%)	84 (92.3%)	7 (7.7%)	142 (91.6%)	13 (8.4%)	0.152
“Should medical professional organizations take a more active role, and have more influence in official health care policy decisions during this major crisis?”	1035 (95.5%)	49 (4.5%)	88 (95.7%)	4 (4.3%)	147 (93.6%)	10 (6.4%)	0.765

### Use of new medications

Only 33% (289/885) of cardiologists and 26% (69/268) of oncologists reported the use of remdesivir or other anti-viral treatments for COVID-19 at their institutions (*p* = 0.035); the use of remdesivir or other antiviral drugs was 27.7% (240/865) in March and 34.4% (129/374) in April. Remdesivir was used by 38% (166/431) in academic vs. 26% (215/826) of private practice and hospital based non-academic affiliated settings (*p* = < 0.001). Overall, reported remdesivir use was 33.8% (323/957) in the US, 21% (18/87) in Europe, and 15% (24/156) in Latin America (*p* = < 0.001) (Fig. 4, Table 3).

Only 15% of both cardiologists and oncologists reported use tocilizumab or other antiinflamatory agents for COVID related myocarditis. The use of tocilizumab changed from 11.9% (100/840) in March to 21.3% (78/366) in April. Tocilizumab was used 22.5% (93/413) in academic vs. only 11.4% (93/817) of non-academic affiliated practices (*p* = < 0.001). Its use was more common in the US at 16.8% (156/927) than in Europe at 11.6% (10/86), or Latin America at 9% (14/155) (*p* = < 0.001).

### Institutional resources and advocacy

Ninety-three percent (451/485) of doctors in academic settings versus 83.9% (765/916) of non-academic affiliated doctors reported to have had in service instruction/guidelines from leadership at their institutions about policies to protect patients, healthcare team and colleagues at the time of this survey (*p* = < 0.001). Eighty percent (391/476) of doctors in academic settings compared to 67.5% (609/914) of non-academic affiliated doctors felt they had support from their institutional leadership to carry on with their duties (*p* = < 0.001). There was strong support for stay in place/lock down policies: 85.7% (826/966) of cardiologists and 86.6% (259/300) of oncologists reported support for national lockdowns/stay in place policies. While support for mandatory lockdown was 84.4% (910/1077) among US cardiologists and oncologists, it was 91.6% (142/155) in Latin America and 92.3% (84/91) in Europe (*p* = 0.152). The overwhelming majority, 95% of all surveyed groups, felt that professional societies should play a larger role in health care policy during the pandemic crisis. In these two health care policy areas there was almost unanimous agreement by physicians in academic medicine or private practice as well as cardiologists and oncologists from all geographic areas (Tables 1, 2 and 3).

## Discussion

The COVID-19 pandemic has been the largest global healthcare crisis of the century and continues to cause a large number of deaths despite the containment efforts [1]. The absence of large-scale testing capabilities with consequent inability to implement successful containment strategy [20], coupled with lack of effective treatment and nonexistent immunization [21], has generated an enormous strain and disruption throughout the world. The emotional and physical toll on healthcare workers, particularly in those areas hardest hit cannot be overstated [22, 23].

We conducted an international survey during the early stage of the pandemic to assess its impact on cardiologists and oncologists in different geographic locations and its effect on scheduling, testing, elective procedures and delay of treatments, as well as the available resources in the work place including PPE, COVID-19 testing, access to telemedicine, and the use of experimental therapies. We also ascertained the degree of existing physician support resources including COVID-19 related institutional guidelines, and physicians’ opinions about endorsement of national stay in place/lockdown policies and their support for active participation of professional societies in health care policy making during a healthcare crisis.

### Geographical impact

We explored regional differences in the impact of this pandemic. There was lower use of telemedicine, less COVID-19 testing, and less use of novel treatments like remdesivir and tocilizumab among Latin America physicians compared US physicians, potentially a reflection of regional economic differences (Table 3). However, other observed differences like less cancer treatment cancelation rates may be secondary to the timing of the pandemic with a later presentation in Latin America. Argentina, a country with strict stay in place/lockdown policies had lower early infection rates than neighboring country Brazil, with no lockdown order, and a rapid growth of COVID-19 cases becoming a world hotspot for the pandemic [1].

More physicians redeployment for COVID-19 care and reduction of specialized care was reported in Europe and Latin America compared to the US, possibly related to different structure of healthcare systems. Interestingly, respondents from Latin America and Europe reported having less institutional leadership support than their US counterparts.

In the UK, cardiologists were redeployed to “COVID-19 wards” and also to provide cardiology support at some of the new purpose-fitted National Health Service (NHS) Nightingale field hospitals (https://www.england.nhs.uk/coronavirus/wp-content/uploads/sites/52/2020/03/specialty-guide-cardiolgy-coronavirus-v1-20-march.pdf). Poland established policies for physicians’ redeployment to other geographic locations. Telemedicine and reimbursement were implemented by the Polish National Health Fund, and recommendation statements for cancer treatment during the pandemic were issued by the Polish Society of Clinical Oncology [24].

### Impact on patient care

The impact of postponed care and its long term consequences are still unknown. Since community spread of SARS-CoV-2 was reported in the US, there were fewer admissions for CVD. A recent retrospective study from 15 US centers reported a 43% reduction in hospitalization rates for acute CV conditions including heart failure, acute coronary syndrome (ACS) and stroke [25, 26]. Likewise, late presentation of ACS and a variety of COVID-19 related “STEMI like” presentations emerged and presented unique challenges in management of these patients [27]. Similarly, delays in cancer treatment can result in devastating consequences. Treatment decisions regarding chemotherapy, immunotherapy, surgery and radiation, were all impacted by the shift of resources during the peak of the COVID-19 pandemic [28]. Unique strategies to try to mitigate risk without compromising outcomes were implemented [29]. In this survey, the reported cancellation of diagnostic and therapeutic procedures, greater among CV patients than cancer patients, points toward a potential future burden to the healthcare system for non-COVID patients due to delayed or deferred care. The discrepancy in canceled testing and procedures between cardiology and cancer patients likely reflects the more time sensitive nature of cancer treatment. In general cancer therapy in the US, Canada and the UK slowed down but continued even in the worst weeks of the COVID-19 pandemic [30, 31].

Similarly, cardiologists were asked to modify the scope of their specialized practice to help with acute patient care during the initial phase of the pandemic more often than oncologists. This may be explained by the acuity of COVID-19 requiring cardiology expertise in acute care management. In addition, the pandemic crisis transiently reduced cardiology utilization of specialty procedures and surgeries. This trend may have already decreased in the US, Canada and others with the re-introduction of elective non COVID-19 related CV care [32].

### Available resources to physicians: PPE, telemedicine, novel therapeutics

The lack of PPE was a significant concern for cardiologists and oncologists with only 74.5% of doctors in academic setting and 67% of private practice/hospital-based doctors feeling they had adequate protection. Similarly, in a recent survey amongst cardiovascular fellows in training (FITs) in the US, only 51% reported wearing N95 masks for all COVID-19 patients, and 41% felt uncomfortable with the PPE recommendations at their institutions [33]. The implications of these findings are significant beyond the effect on doctors themselves, since this perceived lack of safety [15, 16] may impact the doctors’ ability to balance the risk of contagion versus the risk of delayed care.

Telemedicine was infrequently used in most clinical settings prior to the current pandemic [34]. However, the use of telemedicine with existing and new platforms were rapidly adopted during the COVID-19 pandemic as a means of reducing risk of infection [35]. Telemedicine was used more frequently among April than March respondents, and it was more commonly used in academic settings. Although telemedicine was widely utilized in the US, Canada and UK, many countries currently lack a regulatory framework to integrate and reimburse telemedicine services [36]. Telemedicine may improve access to specialty care in small communities and rural areas and may play a critical role in the ability to monitor short and long-term CV complications of cancer treatment. However, the use of this technology may also exacerbate health care disparities. In a recent study with 2940 patients, those with poor socioeconomic status, older age, and non-English speaking had less access to care via telemedicine, particularly video telemedicine [37].

With regard to COVID-19 novel and investigational treatment, we assessed the utilization rate of two of the most promising although yet unproven treatments at the time of the survey. Remdesivir, an inhibitor of the viral RNA dependent RNA polymerase with activity against SARS-COV and Middle East Respiratory syndrome (MERS-COV), has emerged as a potentially useful treatment for patients with advanced COVID-19 [38–40]. Tocilizumab can effectively block the IL-6 signal transduction pathway and could conceptually become an effective drug for patients with severe COVID-19 since significant morbidity and mortality in the late phase of disease is attributed to severe cytokine release storm (CRS) [41, 42]). This drug is also familiar to cardio-oncologists since it is utilized for treatment of CAR-T cell therapy related CRS [41, 42]. The low utilization rate of both remdesivir and tocilizumab reflects the struggle to manage this very sick population given the lack of established and available treatments. Recent promising new data on the use of remdesivir on patients with severe COVID-19 infection [43, 44], may increase its use and availability. This survey did not inquire if these drugs were used off label or as part of a clinical trial.

### Physicians input on Health care policy

One of the main goals of our survey was to garner the opinion of cardiologists and oncologists about the role of government and professional societies during a major health care crisis, a topic that is poorly represented in the medical literature. There was very strong support amongst cardiologists and oncologists for having national health policies in place (e.g. shelter in place) and a need for a clear and coordinated national response to the pandemic. Worldwide, different responses by various governments and health care systems resulted in different outcomes, likely multifactorial given different demographics, cultural habits, and resources of each country [45, 46]. For instance, late responses and failure by governments to act early with containment efforts on the pandemic were associated with widespread and subsequent catastrophic consequences [47]. The presence of a coordinated, unified, early response during future crisis may allow both medical leadership and frontline healthcare workers to perform their duties with less uncertainty and better structured support. Indeed, there was an almost unanimous agreement (95%) among all respondents from all groups in all locations about the imperative need of a very strong participation of the respective professional societies in decision making in public health, particularly during a global health crisis. The active involvement and input by professional medical societies in health care policies may be the instrument that facilitates data driven policies and subsequent better outcomes. Medical societies in many countries including the ACC, ASCO and others have published documents to help providers with health care policies and guidelines [48–52]. This survey suggests physicians’ support and demand such active involvement from professional societies.

#### Study limitations

The responses reflect primarily those of physicians. Many of the addressed issues, including PPE or support for patient care may not reflect those of other healthcare workers.

This survey had a good response from a large number of cardiologists and oncologists from different locations involving both academic and nonacademic doctors. However, there was a strong US response compared to other countries, likely reflecting the larger number of US members in our collaborative cardio-oncology network. Response rates are not available given the multiple sites and organizations that provided this survey utilizing different local platforms, and therefore, a denominator for response rate cannot be established. The survey may have sampling bias and therefore reflect the opinion of physicians more actively involved with medical professional societies. Therefore, the responses cannot be interpreted as representative of all cardiologists, oncologists, nor all institutions at the surveyed locations. Furthermore, since survey questions were not previously validated, respondents’ interpretations may vary, and represent their knowledge and beliefs rather than their institutions’ policies. Moreover, the dynamic nature of this pandemic may have resulted in different responses if this survey was taken at a later time.

However, it highlights the similarities in challenges experienced by physicians working in different environments and points to the need for further awareness, advocacy and collaboration for health care crisis management.

### Advocacy

The health care community remains at the front line of any public health emergency. Professional societies have the mandate and duty to work with their governments to derive data-driven policies aimed at protecting the populations they serve. In order to be better equipped for ongoing and future health care crises, our survey data imply an expectation that our professional societies develop and promote initiatives in the following areas:

1. Promote the use of new technologies and advocate for *permanent* insurance coverage of telemedicine, particularly for vulnerable at-risk patients. Additional research is needed to identify any disparities that may be widened by telemedicine.
2. Have mechanisms in place to expand worldwide production and supply chain of critically needed medical supplies and equipment (such as PPE, ventilators) in a very short period of time.
3. Consolidate algorithms to balance all healthcare needs including pathways for cardiovascular and cancer care during the pandemic with the goal of minimizing delay of essential care.
4. Advocate for close collaboration between medical professional societies and their governments to develop health care policy that will streamline population care during health care crisis.
5. Develop, update, and mandate emergency preparedness in the health care sector to be able to respond in a coordinated manner to a world-wide health crisis in a short period of time.

## Conclusions

This international collaborative survey provides new insight onto the impact of the COVID-19 pandemic on cardiologists and oncologists practices, the lack of resources encountered in many instances, the geographic regional differences and common issues, and the almost unanimous claim by physicians for coordinated central health care policies. Understanding shortcomings and lack of resources experienced during this pandemic and involvement of professional societies in healthcare policy decision making may provide a blueprint for coordination and preparedness for future global healthcare crises.

## Supplementary Information

**Additional file 1.** Link to the Survey: https://www.surveymonkey.com/r/C8ZDYNW.

## Data Availability

The datasets used and/or analyzed during the current studies are available from the corresponding author on reasonable request.

## Abbreviations

COVID-19: Coronavirus disease 2019
CVD: Cardiovascular diseases
SARS CoV-2: Severe acute respiratory syndrome corona virus 2
PPE: Personal protective equipment
ACC: American College of Cardiology
ASCO: American Society of Clinical Oncology
ICOS: International Cardio Oncology Society
CV: Cardiovascular
NHS: National Health Service
