# Supplementary material for: Perspectives on the COVID-19 pandemic impact on cardio-oncology: results from the COVID-19 International Collaborative Network survey
Source: Cardiooncology. 2020 Nov 27;6:28. doi: 10.1186/s40959-020-00085-5 (PMC7691954; doi:10.1186/s40959-020-00085-5)
Supplement: Supplementary file 1 — Additional file 1. Link to the Survey: https://www.surveymonkey.com/r/C8ZDYNW. [file 40959_2020_85_MOESM1_ESM.docx]

| **Cardio-Oncology Survey for ACC State and International Chapters and ICOS Chapters** |
| --- |

Top of Form

Demographics
This information is being collected for statistical reasons only and will not be linked to your answers.

**Question Title**

*** 1. What is your age?**

18-24

25-34

35-44

45-54

55-64

65+

**Question Title**

*** 2. To which gender do you most identify?**

Female

Male

Non-binary

Prefer not to say

**Question Title**

*** 3. What is your specialty?**

Cardiology

Oncology

Internal Medicine

Other

**Question Title**

*** 4. Type of Practice - check all that apply**

Solo

Group Practice

Private

Academic

Hospital Based

Trainee/Resident/Fellow

Urban/Suburban

Rural

**Question Title**

*** 5. In what country do you work?**

**Question Title**

*** 6. If from the United States, in what state or U.S. territory do you currently work?**

**Question Title**

*** 7. Please enter the date you are completing this survey.**

Date / Time

Date

**Question Title**

*** 8. How has COVID-19 impacted your practice? Select all that apply.**

Rescheduling outpatient appointments

Cancellation of most elective procedures

Developed protocols to address patients' anxieties and fears

Decreased use of echo/CT/PET/MR or other imaging modalities

Oncology treatment modified/shortened to balance risk of exposure to COVID-19

**Question Title**

*** 9. Does your institution provide proper protection for all health care workers to the level they are exposed?**

Yes

No

**Question Title**

*** 10. Have timely surgery/chemo/immunotherapy treatments for your patients been delayed since the COVID-19 pandemic started?**

Yes

No

**Question Title**

*** 11. Do you use telemedicine to reduce in-person encounters and health care providers' exposure?**

Yes

No

**Question Title**

*** 12. Have you been asked to reduce your specialized practice to help/contribute to other areas where urgent help is needed?**

Yes

No

**Question Title**

*** 13. Have you had in service instruction/guidelines from leadership at your institution about policies to protect your patients, your team, your colleagues and yourself?**

Yes

No

**Question Title**

*** 14. Do you feel you have adequate support from your institution's leadership to carry on with your duties?**

Yes

No

**Question Title**

*** 15. Do you discuss with all your patients the importance of strict adherence to COVID-19 community behavior as part of your office/clinic encounters?**

Yes

No

**Question Title**

*** 16. Do you have access to COVID-19 testing at your institution or local lab?**

Yes

No

**Question Title**

*** 17. Has your institution used empiric treatment with Remdesivir or other anti-viral treatments for the sickest patients?**

Yes

No

**Question Title**

*** 18. Has your institution used Tocilizumab or other anti-inflammatory agents for COVID-19 related myocarditis?**

Yes

No

**Question Title**

*** 19. In your opinion, should there be a government mandated nationwide quarantine/lock-down to slow down the propagation/transmission of COVID-19?**

Yes

No

**Question Title**

*** 20. Should medical professional organizations take a more active role, and have more influence in official health care policy decisions during this major crisis?**

Yes

No

Done

Bottom of Form
